# Thiazolides Elicit Anti-Viral Innate Immunity and Reduce HIV Replication

**DOI:** 10.1038/srep27148

**Published:** 2016-06-02

**Authors:** Daria Trabattoni, Federica Gnudi, Salomè V. Ibba, Irma Saulle, Simone Agostini, Michela Masetti, Mara Biasin, Jean-Francois Rossignol, Mario Clerici

**Affiliations:** 1 Department of Biomedical and Clinical Sciences L. Sacco, University of Milano, Italy; 2 Don C Gnocchi Foundation, Milano, Italy; 3 Romark Laboratories, L.C., Tampa, Florida, USA; 4 Department of Physiopathology and Transplants, University of Milano, Italy

## Abstract

Nitazoxanide (Alinia^®^, NTZ) and tizoxanide (TIZ), its active circulating metabolite, belong to a class of agents known as thiazolides (TZD) endowed with broad anti-infective activities. TIZ and RM-4848, the active metabolite of RM-5038, were shown to stimulate innate immunity *in vitro*. Because natural resistance to HIV-1 infection in HIV-exposed seronegative (HESN) individuals is suggested to be associated with strong innate immune responses, we verified whether TIZ and RM-4848 could reduce the *in vitro* infectiousness of HIV-1. Peripheral blood mononuclear cells (PBMCs) from 20 healthy donors were infected *in vitro* with HIV-1_BaL_ in the presence/absence of TIZ or RM4848. HIV-1 p24 were measured at different timepoints. The immunomodulatory abilities of TZD were evaluated by the expression of type I IFN pathway genes and the production of cytokines and chemokines. TZD drastically inhibited *in vitro* HIV-1 replication (>87%). This was associated with the activation of innate immune responses and with the up-regulation of several interferon-stimulated genes (ISGs), including those involved in cholesterol pathway, particularly the cholesterol-25 hydroxylase (CH25H). TZD inhibition of HIV-1 replication *in vitro* could be due to their ability to stimulate potent and multifaceted antiviral immune responses. These data warrant the exploration of TZD as preventive/therapeutic agent in HIV infection.

Alinia^®^ (NTZ) is a first-in-class thiazolide (TZD) with an *in vitro* activity against parasites, anaerobic bacteria, and viruses^1^,^2^,^3^. This compound is currently approved in the United States in the treatment of diarrhea caused by *Cryptosporidium parvum* and *Giardia intestinalis*^4^,^5^. It is also approved in Latin America, Egypt, India and Bangladesh for the therapy of a broad range of intestinal protozoan and helminthic infections, and is available upon Emergency Use Authorization in Canada, Australia, Japan and the European Union. NTZ and its active metabolite tizoxanide (TIZ) also inhibit the replication of a broad range of RNA and DNA viruses, and NTZ was shown to be effective in clinical trials against diarrhea caused by rotavirus and norovirus, uncomplicated influenza A and B, as well as hepatitis B (HBV) and C (HCV) infections^6^,^7^,^8^,^9^,^10^,^11^,^12^. A large number of thiazolides closely related to the chemical structure of NTZ have been recently synthesized and tested for their anti-viral activity. RM-5038 in particular, whose active circulating metabolite is known as RM-4848, was selected for complete development due to its efficacy against *C. parvum*^11^,^12^,^13^. The wide spectrum of thiazolides activity was recently confirmed by *in vitro* data indicating an effect of these compounds against HIV. Thus: 1) the association between reverse transcriptase inhibitors (RTI) and NTZ is effective against HIV-1 replication^14^, and 2) NTZ decreases HIV-1 replication in monocyte-derived macrophages (MDM)^15^.

We have recently shown that TIZ and RM-4848 up-regulate a number of genes involved in the TLR- and type I IFN signal transduction pathways [Trabattoni D. and Gnudi F. Personal Communication]. In particular, thiazolides increased the expression of TLR7 and 8, molecules that sense viral-derived components and trigger immune responses against RNA and DNA viruses *via* the induction of type I IFNs. Type I IFNs are essential antiviral proteins that activate a wide array of effectors collectively indicated as IFN-stimulated genes (ISGs). Many of these ISGs inhibit viruses at particular stages of their life cycle^16^,^17^,^18^,^19^,^20^ and were shown to be upregulated in HIV-1 exposed seronegative individuals (HESN)^21^,^22^.

Natural resistance to HIV-1 is thought to result from a favorable genetic and immunologic milieu that, if reproduced in individuals that are not naturally gifted by such favorable profile, could result in induced resistance to this virus^21^,^22^. TLR7 and 8 stimulation, in particular, was shown to elicit strong innate immune responses and the transcription of ISGs with antiviral properties in HESN^23^. Because *in vitro* exposure of PBMCs to thiazolides stimulates the activation of an immune profile that resembles that observed in HESNs, and NTZ was shown to have an antiviral activity that synergizes with that of other drugs, we verified whether *in vitro* susceptibility to HIV infection could be modulated by TIZ or RM-4848.

## Results

### Evaluation of TZDs cytotoxicity in HIV-1-infected cells

**Figure 1:**
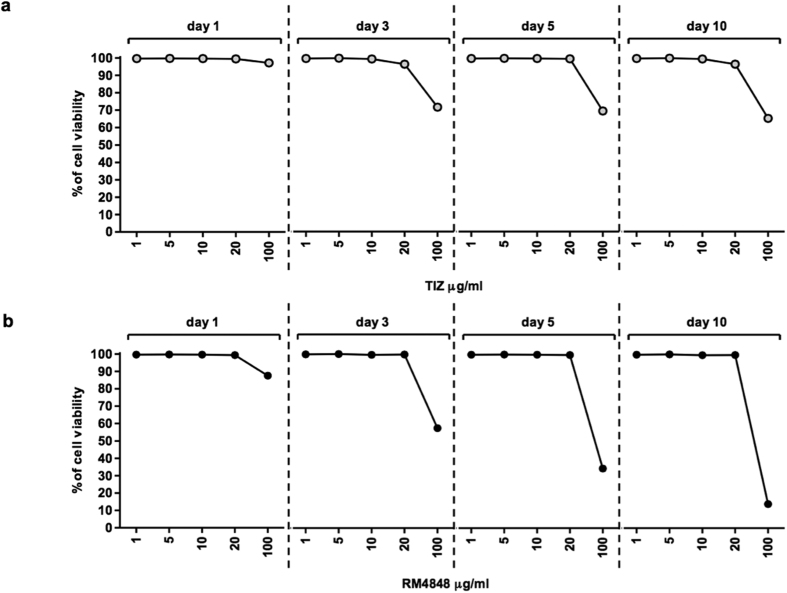
Percentage of cell viability after culture with different doses of TIZ (1, 5, 10, 20 and 100 μg/ml) (**a**) or RM4848 (1, 5, 10, 20 and 100 μg/ml) (**b**) at different time-points (1, 3, 5 and 10 days) assessed using an MTT assay.

Cell viability was analyzed in HIV-1-infected cells cultured in the presence of different doses of TIZ or RM-4848 (1, 5, 10, 20 and 100 μg/ml) by MTT assay. Results indicated that, with the exception of the highest dose, neither TIZ nor RM-4848 alter the viability of PBMCs (Fig. 1a,b respectively). To perform all the experiments a dose of 10 μg/mL of compounds was chosen based on preliminary results showing that concentrations lower than 20 μg/mL are sufficient to maintain pharmacological properties without affecting cell viability [Trabattoni D., Gnudi F. Personal Communication].

### Antiviral activity of TZDs against HIV-1_BaL_ infection *in vitro*

**Figure 2:**
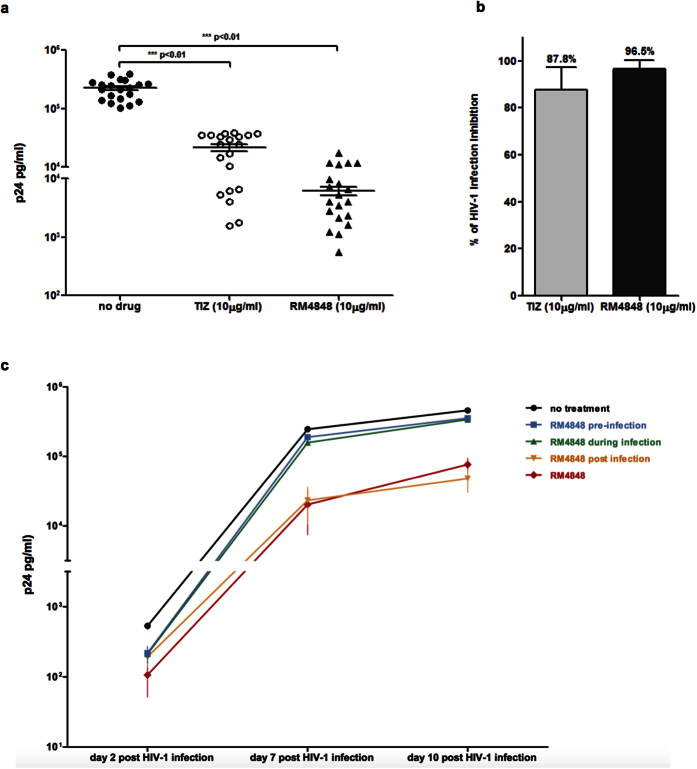
(**a**) p24 viral antigen levels 10 days post-infection in the supernatant of HIV-1 infected PBMCs treated with TIZ (10 μg/mL) or RM-4848 (10 μg/mL). Results obtained with PBMC of 20 different healthy donors are shown. Median values and statistical significance is presented. (**b**) Inhibition (%) of viral replication in PBMC of 20 different healthy donors infected *in vitro* with HIV-1 in the presence of TIZ- (10 μg/mL) or RM-4848- (10 μg/mL). Median values and statistical significance is presented. (**c**) p24 viral antigen levels in PBMCs infected with HIV-1 in the presence of RM-4848 throughout infection (RM-4848)(10 μg/mL) or in medium alone (no treatment). In addition to these two situations, results of 3 other experimental conditions are shown: 1) PBMCs treated with RM-4848 before being infected (pre-infection); 2) RM-4848 present during the 24 hours infection period (during infection) and then washed away; 3) RM-4848 added 24 hours after infection (post infection). Mean values ± standard errors are shown.

*In vitro* infection of PBMCs with HIV-1 was significantly and consistently reduced by TIZ and RM-4848; this effect was reproduced in all the experiments (n = 20) in which cells from randomly selected donors not carrying genetic markers known to reduce susceptibility to HIV infection were used (see Methods). The overall inhibition of HIV-1 replication *in vitro* was 87.8% for TIZ and 96.5% for RM-4848 (p-value < 0.01 *vs*. medium alone) at a 10μg/mL concentration (Fig. 2a,b).

The antiviral effect of RM-4848 was further examined by adding this compound to cell cultures at different time-points: 1) for two days before HIV-1 infection and then washed away (pre-infection); 2) during the 24 hours infection period infection followed by washout; or 3) 24 hours after infection and untill the end of the cell culture period. Cells infected with HIV-1 in the presence of RM-4848 throughout infection (RM-4848) (10 μg/mL) or in medium alone (no treatment) were considered as controls. The strongest inhibition of viral replication was observed when RM-4848 was added to the cultures at the time of infection, or one day after infection (Fig. 2c).

### Modulation of type I IFN pathway by TZDs

**Figure 3:**
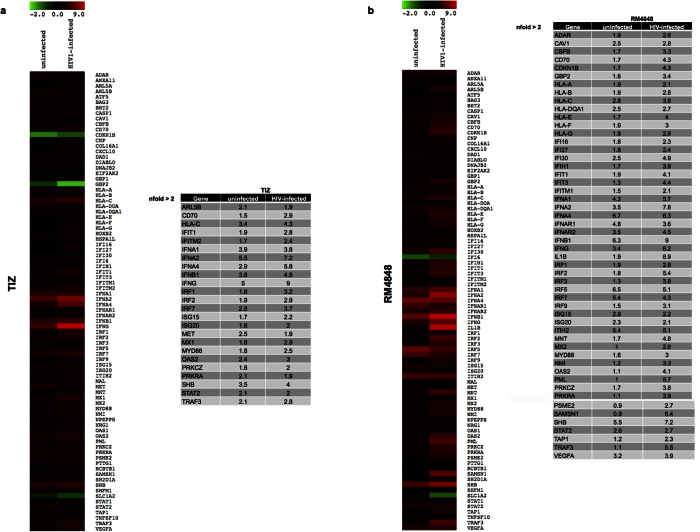
mRNA expression of 84 genes that are part of the type I IFN signal transduction pathway has been assessed by real-time quantitative RT-PCR, calculated relative to five housekeeping genes and shown as fold-change expression from the untreated sample. Gene expression (nfold) is shown as a color scale from green to red (−2 to +9) (MEV multiple experiment viewer software). (**a**) Uninfected and HIV1-infected (day 7 post infection) PBMCs cultured in medium containing TIZ (10 μg/mL); (**b**) Uninfected and HIV1-infected (day 7 post infection) PBMCs cultured in medium containing RM-4848 (10 μg/mL). Only targets showing at least a 2-fold modulation were considered significative and shown in tabs.

The ability of thiazolides to up-regulate type I IFN and ISG expression levels in this model of *in vitro* HIV-1-infection was analyzed in PCR array that screens simultaneously 84 genes involved in the type I IFN pathway. Exposure of PBMCs to TIZ resulted in an increased expression of a number of IFNs (IFNα1, IFNα2, IFNα4, IFNβ1, IFNγ) and ISGs (ARL5B, IRF7, MET, OAS2, PRKRA, SHB, STAT2, TRAF3), as well as of HLA-C (Fig. 3a). Upon HIV-1 infection this effect was potentiated and amplified, and different genes were strongly up-regulated. These included IFNs (IFNα1, IFNα2, IFNα4, IFNβ1, IFNγ), ISGs (IFIT1, IFITM2, IRF1, IRF2, IRF7, ISG15, ISG20, MX1, MyD88, OAS2, PRKRCZ, SHB, STAT2, TRAF3), HLA-C and CD70, an important immunoregulatory receptor (Fig. 3a). In all conditions the effect of RM-4848 was more robust than that of TIZ (Fig. 3b).

### Analysis of anti-HIV-1 human response pathway

**Figure 4:**
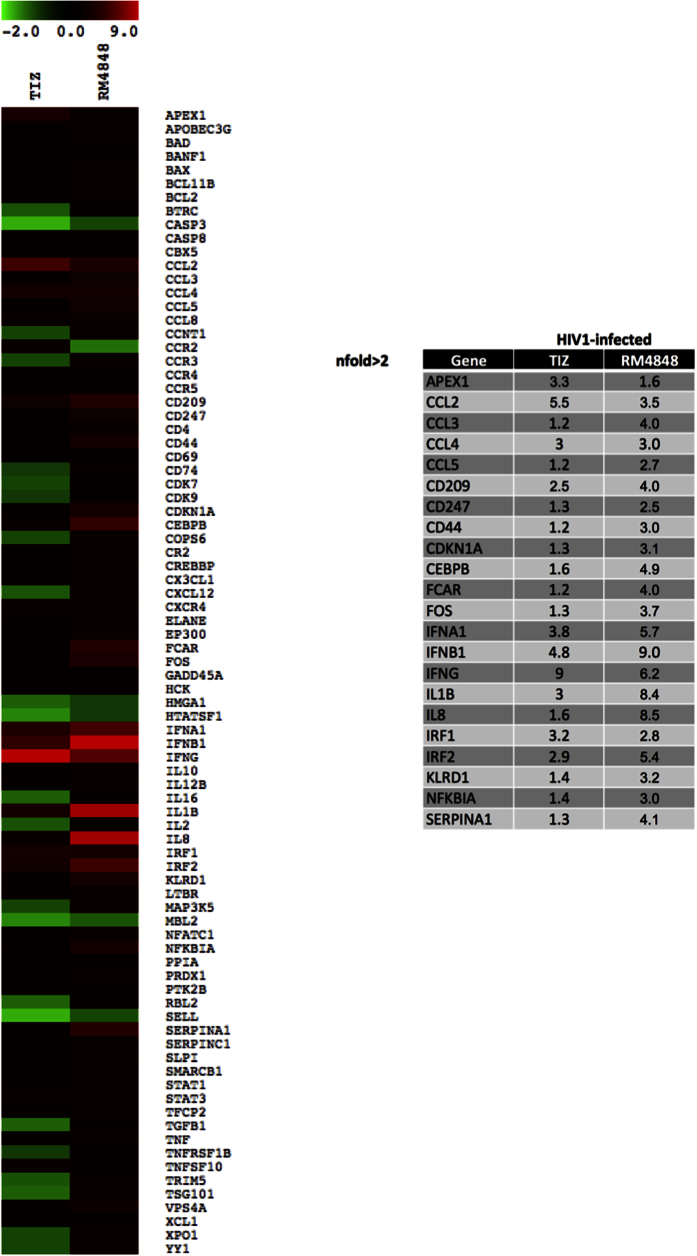
mRNA expression of 84 genes that are part of the HIV-1 life cycle and known to directly obstacle HIV infectivity has been assessed by real-time quantitative RT-PCR, calculated relative to five housekeeping genes and shown as fold-change expression from the untreated sample. Gene expression (nfold) is shown as a color scale from green to red (−2 to +9) (MEV multiple experiment viewer software). Results were obtained in HIV1-infected PBMCs at day 7 post infection, cultured in medium containing TIZ (10 μg/mL) or RM-4848 (10 μg/mL). Only targets showing >2-fold modulation are considered significant and are shown in tab.

Results above show that thiazolides induce the up-regulation of genes such as IRF1, OAS2 and PRKRA known to obstacle HIV infectivity. To analyze more in depth whether the anti-HIV effects of TZDs stem from the modulation of cellular factors involved in the HIV-1 life cycle, we used an anti-HIV-1 human response pathway array that screens for the expression of such factors. Results indicated that the *in vitro* HIV-inhibitory properties of TZDs are only partially explained by the modulation of HIV-1 life cycle genes. Also in these experiments the antiviral activity of RM-4848 was more potent than that of TIZ (Fig. 4).

### TZDs modulate cytokine and chemokine production in HIV-1 infected PBMCs

**Figure 5:**
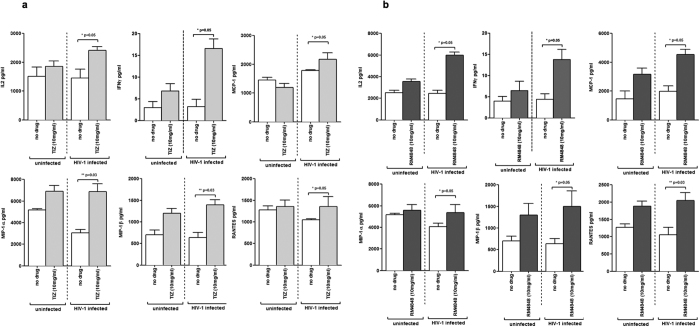
Cytokines (IL-2, IFNγ) and chemokines (MCP-1, MIP-1α, MIP-1β, RANTES) production (pg/ml) by PBMCs of healthy donors. PBMCs were either uninfected (white bars) or HIV-1-infected (grey bars) and were cultured in medium alone (no drug), or in medium containing either TIZ- (10 μg/mL) (**a**) or RM-4848- (10 μg/mL) (**b**). Mean values 7 days post-infection ± standard errors and statistical differences are shown.

Cytokine and chemokine production by HIV-infected PBMCs in the presence of thiazolides was measured next. Results showed that IFNγ and IL-2 production was significantly increased in HIV-1 infected cells by both TIZ and RM-4848 (p < 0.05 *vs*. medium alone). Even more interestingly, the production of those CC chemokines known to be endowed with potent anti-HIV activities (MCP-1, MIP1-α, MIP1-β, RANTES) was modulated by both TIZ (Fig. 5a) and RM-4848 (Fig. 5b) (p < 0.05 *vs.* medium alone). These results suggest that thiazolides obstacle viral entry and/or reduce the infection process of target cells.

### TZDs up-regulate ISG involved in cholesterol metabolism

**Figure 6:**
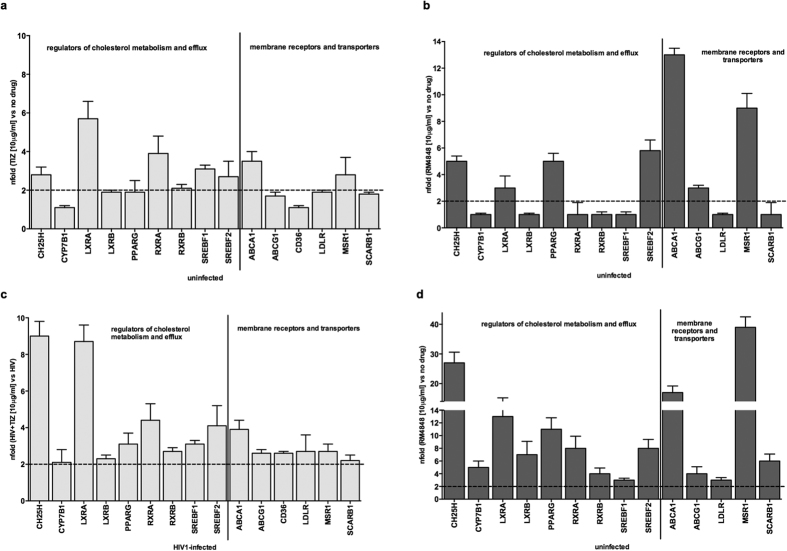
mRNA expression of genes involved in cholesterol metabolism and efflux in PBMCs of healthy donors. (**a**) Uninfected PBMCs cultured in medium containing TIZ (10 μg/mL); (**b**) Uninfected PBMCs cultured in medium containing RM-4848 (10 μg/mL); (**c**) HIV-1–infected PBMCs (day 7 post infection) cultured in medium containing TIZ (10 μg/mL); (**d**) HIV-1–infected PBMCs (day 7 post infection) cultured in medium containing RM-4848 (10 μg/mL). Gene expression genes is assessed by quantitative RT-PCR and shown as fold-change expression from the untreated sample. Only targets showing >2-fold modulation are considered significant. Mean values ± S.D. and p values are indicated.

Since thiazolides up-regulate type 1 IFN and ISGs, and because ISGs involved in the metabolism and the efflux of cholesterol were recently shown to have a potent antiviral activity, we verified whether these compounds may also affect cholesterol metabolism, and in particular CH25H expression. Results obtained in uninfected PBMCs confirmed this hypothesis, showing an up-regulation of genes involved in cholesterol metabolism and efflux and of different membrane receptors and cholesterol transporters both by TIZ (Fig. 6a) and RM-4848, with the effects of this latter compound once again being more potent (Fig. 6b).

These effects were more pronounced upon HIV-1 infection as TIZ and, mostly, RM-4848 up-regulated the expression of proteins (CH25H, CYP7B1, LXRα, LXRβ, PPARγ, RXRα, RXRβ SREBF1, and SREBF2) and membrane receptors (MSR1, SCARB1, LDLR, ABCG1 and ABCA1) involved in cholesterol metabolism and efflux (Fig. 6c,d). CH25H, in particular, an enzyme recently shown to play a role in preventing viral spread to target cells HIV^20^, was robustly (>12 fold) up-regulated by thiazolides in HIV-1-infected cells.

### Thiazolides upregulation of Interferon-Inducible Cholesterol-25-Hydroxylase (CH25H)

**Figure 7:**
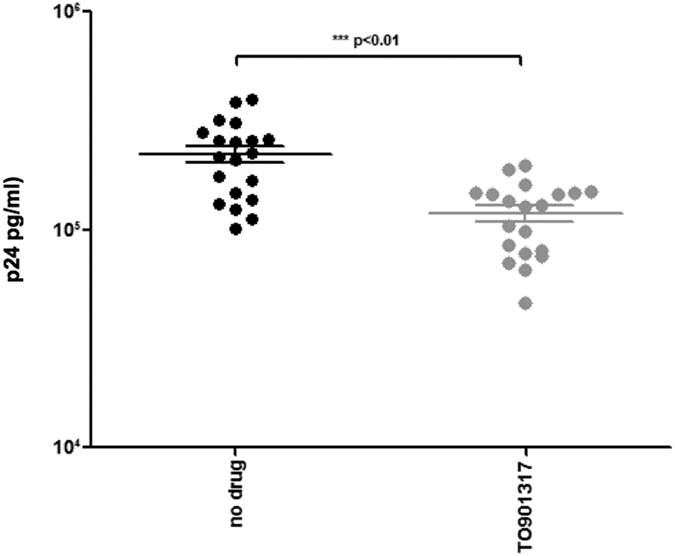
p24 viral antigen concentration in HIV-1_-_ infected PBMCs cultured in medium alone (no drug) or in the presence of TO-901317 (1 μM), an LXR agonist. Results were obtained with PBMCs of 20 different healthy donors 10 days post-infection. Mean values ± standard errors and statistical significance are shown.

Activation of CH25H results in an enzymatic reaction that generates 25-HC, a natural oxysterol that mediates the efflux of cholesterol from cells and ligates the liver X receptor (LXR). To analyze possible correlations between genes involved in cholesterol efflux and *in vitro* inhibition of HIV-1 infection we stimulated HIV-1-infected cells with a synthetic agonist of LXR (TO-901317) and assessed viral infectivity. Results showed that agonist-mediated activation of LXR resulted in a significant decrease of cell infection (52%; p < 0.01) (Fig. 7), suggesting that the protective effects of thiazolides on HIV-1 infectivity are at least partially achieved through the modulation of cholesterol efflux mechanisms.

## Discussion

The vast spectrum of antimicrobial activity of thiazolides was recently enriched by results indicating that these compounds are effective in reducing *in vitro* HIV infection^14^,^15^. We investigated the immunologic basis of this effect and show herein that incubation of PBMCs with thiazolides results in the secretion of type 1 cytokines and of CC chemokines, as well as the upregulation of type I IFN genes and interferon-stimulated genes (ISGs). This effect was potentiated upon exposure of cells to HIV, and is associated with a significantly and reproducible reduction of HIV infectiousness of lymphocytes isolated from healthy individuals.

These results indicate that TZDs hamper HIV-1 infection of target cells and reduce viral replication using different and possibly overlapping modalities. Thus: 1) CC chemokines, whose production is stimulated by TZDs, impede the infection of target cells by CCR5 tropic viruses^24^,^25^, 2) Type 1 cytokines and cell-mediated immune responses contrast HIV-1 replication^26^,^27^, and 3) the stimulation of the transcription rate of genes endowed with generic antiviral effects, or know to have a specific ability to obstacle HIV-1 replication by TZDs, likely contributes to the anti-HIV properties of these compounds. Amongst the genes with generic antiviral effects that are upregulated by TZDs the activity of these compounds on: 1) PRKRA, a protein kinase activated by dsRNA that inhibits cellular mRNA translation and the synthesis of viral proteins^18^,^26^, 2) ADAR, an enzyme that destabilizes double-stranded RNA through conversion of adenosine to inosine^28^, 3) OAS2, which activates RNAses and induces the degradation of viral RNA and the inhibition of protein synthesis^29^, and 4) STAT2, a protein that activates the transcription of ISG and drives infected cell in an antiviral status^30^, is likely very important. Within genes that are upregulated by thiazolides and have a direct anti HIV-1 activity, IRF-1 a protein required for efficient HIV-1 replication^31^, and MX2 which acts as an inhibitor of HIV-1 infection by blocking viral replication at a late post-entry step, could play pivotal roles^32^,^33^. Notably, recent observations showed that IFN effectors work in cooperation to achieve a fully functional antiviral state, i.e., combination of different ISGs expressed together, such as what is observed in TZDs-treated cells, results in an ampler magnitude of antiviral activity than either gene alone^34^. These results would explain the greater efficacy of RM-4848 compared to TIZ in reducing HIV-1-infection, given the fact that RM-4848 results in the upregulation of a broader range of ISGs compared to what is seen in cells exposed to TIZ.

The observation that CH25H, an oxysterols-producing enzyme, is up-regulated by TIZs, is particularly intriguing. Viral infections themselves results in the rapid induction of CH25H and the consequent production of 25-HC via STAT1, a type I IFN effector. Interestingly, 25HC inhibits the growth of a wide range of enveloped viruses by inducing structural changes in the cellular membrane that impair viral entry at the virus-cell fusion step^20^. Our data show that thiazolides indeed up-regulate the expression of a number of genes that are part of cholesterol metabolism. Thus, the transcription factors of the sterol regulatory element–binding protein (SREBP) family, SREBP1 and SREBP2 are involved in the regulation of cholesterol biosynthesis^35^. Macrophage scavenger receptor-1 (MSR-1)^36^ and the LDL receptor (LDL-R)^37^ are responsible for the influx of lipids into macrophages. Finally, liver X receptor (LXR) α, and the peroxisome proliferated factor (PPAR) γ coordinate the removal of intracellular cholesterol^36^,^38^. Notably, TZDs also increased the expression of genes that encode lipid-transport proteins, such as the ATP-binding cassettes A1 (ABCA1) and G1 (ABCG1), by which intracellular excess of cholesterol is transported to extracellular acceptors^35^,^39^. The observations that: 1) thiazolides reduce *in vitro* HIV-1 infection; 2) TZDs up regulate LXR; and 3) *in vitro* HIV-1 infection of immune cells is significantly reduced by LXR agonists allow the speculation that the antiviral effects of these compounds are at least partially mediated by their ability to modulate proteins within the cholesterol metabolic pathway. Notably, this is a speculative hypothesis that will need to be tested in furhter experiments.

These results indicate that TZDs are endowed with strong and multifaceted antiviral activities, and confirm previous *in vitro* observations^14^ suggesting that the use of these compounds might be envisioned in the therapy of HIV infection. In this light, preliminary results from a clinical study carried out in the United States in treatment-naïve HIV-HCV co-infected subjects are interesting. In this pilot study the activity on HIV viral loads (VL) of nitazoxanide (NTZ) alone and in combination with pegylated- interferon and ribavirin (PEG-IFN/RBV) was evaluated. At baseline, HIV VL was available in 65 cases; after 16 weeks of NTZ in combination with PEG-IFN/RBV, HIV VL was undetectable (<20 copies/ml) in 12 of the 14 (86%) patients in whom it could be reanalyzed. Notably, 5 of these 14 individuals were ARV-naïve throughout the study period; median HIV VL log_10_ change in these patients was −1.32; this effect was presumably mediated by NTZ (J. F. Rossignol, Personal Communication). These results should be interpreted with caution not only because of the small sample size and lack of a direct comparator, but also because the subjects had low viral loads at baseline, making the overall change in HIV VL difficult to quantify. These data nevertheless support the suggestion that NTZ may have a clinical benefit in patients infected with HIV.

Results herein show that the mechanism of activity of thiazolides is entirely new and is mediated by the activation of multiple and possibly synergistic mechanisms. Such mechanisms might confer to immune cells isolated from healthy individuals an “HESN-like” status, characterized by a drastic reduction of susceptibility to *in vitro* HIV infection. Additional disease models as well as clinical trials will be needed to elucidate the possible role of thiazolides in novel preventive and therapeutic approaches against HIV-1 infection and, possibly for the therapy of pathologies associated with alterations of the cholesterol metabolism.

## Methods

### Thiazolides

TIZ and RM-4848 were supplied by Romark Laboratories, L. C. (Tampa, Florida) and were suspended in dimethyilsulfoxide (DMSO, Sigma Aldrich, St. Louis, MO, USA) to obtain a 40 μg/ml solution and stored at 2–8 °C until use.

### HIV-1 strains

The laboratory-adapted HIV-1 strain used in the experiments was the R5 tropic HIV-1_BaL_ (courtesy of Drs. S. Gartner, M. Popovic and R. Gallo; NIH AIDS Research and Reference Reagent Program) provided through the EU program EVA centre for AIDS Reagents (NIBSC, Potter Bars, UK). In initial experiments two other HIV-1 strains were used: HIV-1_IIIB_, a subtype B CXCR4-tropic strain, and HIV-1_DU174_, a subtype C CCR5-tropic strain. Because results obtained with HIV-1_BaL_, HIV-1_IIIB and_ HIV-1_DU174_ were always comparable, HIV-1_BaL_ alone was used in the experiments presented in the paper.

### PBMC isolation and HIV-1 infection

Whole blood was collected from 20 healthy volunteers by venipuncture in Vacutainer tubes containing EDTA (Becton Dickinson, Franklin Lakes, NJ), and PBMCs were separated on lymphocyte separation medium (Organon Teknica, Malvern, PA). Subjects expressing genetic markers known to be associated with a reduced susceptibility to *in vitro* HIV infection (CCR5Δ32; HLA-B27 and –B7; CCL3L1 as well as SNPs in APOBEC3G, TRIM5α and MX2) were excluded from the analyses. Healthy volunteers signed a informed consensus, as agreements between the University of Milan and the Vimercate Hospital, (Vimercate, MB, Italy). This protocol was reviewed and approved by the Ethics Committee of the Luigi Sacco University Hospital in Milan.

All the procedures were carried out in accordance with the GLP guidelines adopted in our laboratory. PBMCs (2 × 10^6 ^cells/mL) were re-suspended in medium containing 1 ng/1 × 10^6 ^cells of HIV-1_BaL_ p24 viral input and incubated for 24 hours at 37 °C and 5% CO_2_. Cells were then washed and re-suspended in complete medium with interleukin-2 (IL-2, 15 ng/mL) (Sigma-Aldrich, St. Louise, MO, USA) and phytohemagglutinin (PHA, 7.5 μg/mL) (Sigma-Aldrich) for 3 days. After viability assessment cells were washed and re-suspended in RPMI complete medium with IL-2 (15 ng/ml) and TIZ (10 μg/mL), or RM-4848 (10 μg/ml). 50% of the culture medium was renewed every 3 days. PBMCs were collected for gene expression at day 7 and 10 post-infection. In some experiments the conditions were modified as follows: 1) TZDs added to cell cultures before infection and then washed away; 2) TZDs added to cell cultures at the same time of HIV, left during the infection period (24 hours), and subsequently washed away; 3) TZDs added post-infection and left in the culture wells throughout the 10 days experiment period. Finally, in other experiments, the LXR agonist TO901317 (1 μM/mL) (Sigma-Aldrich) was added to the cultures post HIV-1 infection.

### Evaluation of the toxicity of TZDs

Cells were cultured in RPMI medium (Euroclone, Milan, Italy) supplemented with 20% FBS (Euroclone, Milan, Italy), penicillin and streptomycin, L-glutamine and gentamycin (Euroclone, Milan, Italy) alone or in presence of increasing concentration (0.5 μg/ml, 1 μg/ml, 10 μg/ml, 20 μg/ml and 100 μg/ml) of drugs, diluted in medium culture for 1, 3, 5, 7 and 10 days. Every two days the 50% of medium was removed and replaced with fresh medium containing drugs at the corresponding concentrations. Toxicity of thiazolides was determined by a MTT based assay (Sigma Aldrich, St. Louis, MO, USA) according to manufacturer’s protocol. PBMCs viability were established by dividing the absorbance reading of the formazan by the dry weight of cell cultures. Toxicity were determined by dividing viability of the drug- treated cells by viability of untreated cell control.

### HIV-1 replication

Culture supernatants were collected at day 3, 7 and 10 after infection. p24 concentration was assayed using the Alliance HIV-1 p24 Antigen kit (Perkin Elmer, Boston, USA) following manufacturer’s instructions.

### Cytokine and chemokine measurement

Cytokines and chemokines concentration (IL2, IFNγ, MCP-1, MIP1α, MIP1β and RANTES) was assessed in supernatants of uninfected and HIV-1 infected cells cultures using multiplex sandwich immunoassays (Fluorokine Multi Analyte Profiling Kit; R&D Systems, Minneapolis, MN, USA) according to manufacturer’s protocol. Supernatants were obtained after 7 days of infection.

### RNA extraction and Retro-transcription (RT)

RNA was extracted from 1 × 10^6^ PBMCs by using the acid guanidium thiocyanate–phenol–chloroform method. RNA was dissolved in RNase-free water, and purified from genomic DNA with RNase-free DNase (RQ1 DNase, Promega, Madison, Wisconsin, USA). One microgram of RNA was reverse transcribed into first-strand cDNA in a 20-μl final volume containing 1 μM random hexanucleotide primers, 1 μM oligo dT and 200 U Moloney murine leukemia virus reverse transcriptase (Clontech, Palo Alto, California, USA).

### Real time PCR

cDNA quantification was performed by real-time PCR (DNA Engine Opticon 2; MJ Research, Ramsey, MN). Reactions were performed using a SYBR Green PCR mix (Promega, Fitchburg, WI, USA) and amplified according to the following thermal profile: initial denaturation (95 °C, 15 minutes) followed by 40 cycles of 15 seconds at 95 °C (denaturation) and 1 minute at 60 °C (annealing) and 20 seconds at 72 °C (extension). A Ct value of 40 or higher means no amplification and this value was not included in the calculations. Results were expressed as ΔΔCt and presented as ratio between the target gene and the GAPDH housekeeping mRNA. All the samples were analyzed in triplicate.

### TLR, Type I IFN and HIV-1 human response pathway analysis

TLR, type I IFN and HIV-1 human response pathway were analysed in a PCR array including a set of optimized real-time PCR primer assays (SABiosciences Corporation, Frederick, MD, USA). This approach permits monitoring of mRNA expression of 84 genes related to the different pathways, plus five housekeeping genes. Results were analysed using the SABiosciences online software. Only targets showing >2-fold modulation were considered significant. Experiments were run on the subjects included in the study pooled into distinct groups (medium alone, TIZ or RM-4848) and represent the mean value of the different targets analyzed in each group.

### Statistical analysis

Comparisons between groups were analyzed to evaluate immunological differences. Kruskal & Wallis analysis of variance was performed for each variable; Bonferroni correction was applied to the results. Two-sided p-values were considered. Data analysis was performed using the SPSS statistical package (SPSS Inc. Chicago, IL, USA).

## Additional Information

**How to cite this article**: Trabattoni, D. *et al*. Thiazolides Elicit Anti-Viral Innate Immunity and Reduce HIV Replication. *Sci. Rep.* **6**, 27148; doi: 10.1038/srep27148 (2016).
